# Salivary cotinine concentrations in daily smokers in Barcelona, Spain: a cross-sectional study

**DOI:** 10.1186/1471-2458-9-320

**Published:** 2009-09-03

**Authors:** Marcela Fu, Esteve Fernandez, Jose M Martínez-Sánchez, José A Pascual, Anna Schiaffino, Antoni Agudo, Carles Ariza, Josep M Borràs, Jonathan M Samet

**Affiliations:** 1 Tobacco Control & Research Unit, Institut Català d'Oncologia (ICO-IDIBELL). Av. Gran Via de L'Hospitalet 199-203, 08907 L'Hospitalet de Llobregat (Barcelona), Spain; 2 Department of Clinical Sciences, School of Medicine, Campus de Bellvitge, Universitat de Barcelona. Pavelló de Govern, Feixa Llarga s/n, 08907 L'Hospitalet de Llobregat (Barcelona), Spain; 3 Bioanalysis Research Group, Neuropsychopharmacology Programme, IMIM-Hospital del Mar. Parc de Recerca Biomèdica de Barcelona, Doctor Aiguader 88, Barcelona, Spain; 4 Department of Experimental and Health Sciences, Universitat Pompeu Fabra. Parc de Recerca Biomèdica de Barcelona, Doctor Aiguader 88, 08003 Barcelona, Spain; 5 Ajuntament de Terrassa. Crta. de Montcada 596, 08223 Terrassa, Spain; 6 Nutrition, Environment & Cancer Unit, Institut Català d'Oncologia (ICO-IDIBELL). Av. Gran Via de L'Hospitalet 199-203, L'Hospitalet de Llobregat (Barcelona), Spain; 7 Evaluation & Intervention Methods Unit, Agència de Salut Pública de Barcelona (ASPB). Pl. Lesseps 1, 08023 Barcelona, Spain; 8 CIBER de Epidemiología y Salud Pública (CIBERESP). Parc de Recerca Biomèdica de Barcelona, Doctor Aiguader 88 1a Planta, 08003 Barcelona, Spain; 9 Pla Director d'Oncologia. Av. Gran Via de L'Hospitalet 199-203, 08907 L'Hospitalet de Llobregat (Barcelona), Spain; 10 Department of Preventive Medicine, USC Institute for Global Health, Keck School of Medicine USC, California, USA; 11 Norris Comprehensive Cancer Center. 1441 Eastlake Avenue, Room 4436, MS 44; Los Angeles CA 90033, USA

## Abstract

**Background:**

Characterizing and comparing the determinant of cotinine concentrations in different populations should facilitate a better understanding of smoking patterns and addiction. This study describes and characterizes determinants of salivary cotinine concentration in a sample of Spanish adult daily smoker men and women.

**Methods:**

A cross-sectional study was carried out between March 2004 and December 2005 in a representative sample of 1245 people from the general population of Barcelona, Spain. A standard questionnaire was used to gather information on active tobacco smoking and passive exposure, and a saliva specimen was obtained to determine salivary cotinine concentration. Two hundred and eleven adult smokers (>16 years old) with complete data were included in the analysis. Determinants of cotinine concentrations were assessed using linear regression models.

**Results:**

Salivary cotinine concentration was associated with the reported number of cigarettes smoked in the previous 24 hours (*R*^2 ^= 0.339; p < 0.05). The inclusion of a quadratic component for number of cigarettes smoked in the regression analyses resulted in an improvement of the fit (*R*^2 ^= 0.386; p < 0.05). Cotinine concentration differed significantly by sex, with men having higher levels.

**Conclusion:**

This study shows that salivary cotinine concentration is significantly associated with the number of cigarettes smoked and sex, but not with other smoking-related variables.

## Background

Nicotine, the main alkaloid of tobacco, is responsible for its addictive effect. It is readily absorbed from tobacco smoke, and its concentration rises over 6-8 hours during the day in regular smokers [1]. About 70 to 80% of nicotine is metabolized to cotinine [2]. As the primary metabolite of nicotine, cotinine has been widely used as a specific biomarker of tobacco exposure because its half-life in the body (12-20 hours) is longer than that of nicotine (3-4 hours) [1,2]. Cotinine in biological materials is suitable for assessment of doses over short periods of time (from 1 to 10 days, in urine, plasma, or saliva) or longer periods (weeks or months, in hair or nails). Consequently cotinine concentration is feasibly used as a biomarker in epidemiological studies [3-5].

Cotinine measurements have been used to describe and compare patterns of tobacco consumption in smokers in different countries to establish if addiction and smoking patterns vary across populations [6-8]. These studies show that the number of cigarettes smoked is the main determinant of salivary cotinine concentrations [9]. These studies have been conducted in selected samples from a variety of countries at different stages of the tobacco epidemic, such as China, Mexico, Brazil, and Poland [9,10]. However, there is scant information about the relation between cotinine measurements and smoking patterns in samples from the general population.

Spain is currently in an advanced stage of the tobacco epidemic [10,11]. Data from the 2006 Spanish National Health Interview Survey show prevalence rates of daily smokers of 31.6% and 21.5% in adult men and women, respectively [12]. In men, a steady increase in smoking occurred during the first half of the twentieth century, reaching a peak prevalence rate of 59.1% in the nineteen-seventies. This peak was followed by a decade of stabilization and a continued decrease of smoking until the present. Uptake of smoking in women was delayed, with a prevalence rate of 5% through the nineteen-seventies. This was followed by a substantial increase throughout the next two decades (22.5% by 1995), which only recently stopped [11,13,14].

An understanding of cotinine concentration and smoking patterns at the population level is potentially useful to design suitable strategies for cessation. The aim of this study is to describe and characterize the distribution of salivary cotinine concentration in a representative sample of adult (>16 years old) daily smokers in Barcelona, Spain.

## Methods

### Study design and subjects

We conducted a cross-sectional study among the general population of Barcelona, Spain, between March 2004 and December 2005. A representative random sample by age, sex, and district was drawn from the official 2001 population census of Barcelona, a reliable source of population-based information. To detect a difference in cotinine concentration in smokers of 50 ng/ml (with a mean value of 500 ng/ml and a standard deviation of 200 ng/ml), with an alpha of 5% and a beta of 10% (statistical power of 90%), we estimated that a sample size of 337 smokers would be needed. Considering a 27% prevalence of smoking from the 2001 Health Survey of Barcelona, we estimated a needed sample size of 1560 people, taking into account smokers and non-smokers. In cases of non-response, substitution by persons of the same sex within the 5-year age group and residing in the same district was allowed according to protocol.

Smoking status, secondhand smoke exposure as well as demographic information were obtained by questionnaire, and a saliva specimen was collected to determine cotinine concentration. The research and ethics committee of the Bellvitge University Hospital approved the study protocol, and informed consent was obtained from all participants. The procedure was as follows: a personal letter was sent to eligible participants, and trained interviewers contacted the subjects (or a proxy for children) at home and informed them about the study. Participants signed the consent form, answered a questionnaire, and provided a saliva specimen at home. The study ended in December 2005 with a new Spanish law banning smoking in public places and enclosed workplaces coming into effect in January 2006 [15]. We expected changes in smoking behavior after this date (number of cigarettes smoked by smokers and passive exposure levels in nonsmokers) and hence 315 selected participants were not approached. By the end of the study, 1245 subjects had been interviewed (participation rate of 79.8% from the initial sample drawn). The study design allowed replacement of the index person by another person of the same sex, 5-year age group, and district of residence. In 49.3% of cases the first selected index person was interviewed; 24.4% of first substitutes were interviewed; and 26.3% of second or subsequent substitutes were interviewed. The final sample interviewed included 285 daily smokers (at least 1 cigarette per day), 62 occasional (non-daily) smokers, 354 ex-smokers, 525 never-smokers, and 19 people were less than 17 years of age. The present report is based on adults who were daily smokers.

### Measures

#### Questionnaire

We obtained information on demographics, and levels of secondhand smoke exposure at home, work or study centre, and during leisure time. Detailed information was also collected on self-reported smoking for smokers: number of cigarettes smoked daily, number of cigarettes smoked during the previous 24 and 48 hours, duration of smoking, brand of cigarettes smoked most often, brands of cigarettes smoked during the survey day and in the previous day, use of cigarettes with filter tips, depth and frequency of inhalation, use of other tobacco products, and use of nicotine gum or patches.

#### Body mass index

We measured participants' weights and heights using a standardized protocol (with an electronic portable scale and a tape measure). Body mass index (BMI) was computed as weight/squared height (kg/m^2^) and stratified using standard categories of BMI (underweight: <18.50, normal: 18.50-24.99, overweight: 25.00-29.99, and obese: ≥30.00 kg/m^2^).

#### Saliva specimen

A standardized protocol for saliva collection was used. Participants were asked to rinse their mouths and then suck a lemon-flavored candy (Smint^®^) to stimulate saliva production. They were asked first to spit out a small amount of saliva, and then to spit about 8 ml into a polypropylene test tube. The specimens were kept at 4°C and then frozen at -20°C in 3 ml aliquots for transport in dry ice to the Bioanalysis Research Group of the Municipal Institute for Medical Research (IMIM-Hospital del Mar). Cotinine concentration in nanograms per milliliter (ng/ml) was determined by gas chromatography, with detection by mass spectrometry (GC/MS) [16,17]. With this technique, cotinine concentration can be quantified as low as 1 ng/ml (limit of detection: 0.3 ng/ml; quantification error <15%).

### Data analyses

#### Sample exclusions

Of the 285 current adult daily smokers, 7 were excluded from the analysis either because they did not provide a saliva specimen or that cotinine determination was not possible (i.e., insufficient sample). We included only cigarette smokers, hence 53 people who smoked other tobacco products (mainly cigars and roll tobacco) were excluded, as were 2 subjects who used nicotine gum or nicotine patch for cessation. Additionally, 12 people were excluded because their cotinine concentrations were too high in relation to the self-reported consumption, that is, over 35 ng/ml per one cigarette smoked. This level of cotinine represents the maximum level of absorption per one cigarette smoked, assuming that the typical cotinine concentration of 12 ng/ml per cigarette is equivalent to the usual absorption of 1 mg of nicotine per cigarette, and that a cigarette smoker can absorb up to 3 mg of nicotine per cigarette with very intense smoking [9]. The final sample for analysis consisted of 211 current daily smokers.

#### Variables

The outcome variable was salivary cotinine concentration (ng/ml). Potential modifiers of the relation between cotinine concentration and the number of cigarettes smoked in the last 24 hours included individual characteristics (sex, age, educational level, BMI), type of tobacco (use of regular or non-regular cigarettes [light, ultralight, mentholated, low nicotine yield], use of blond or black tobacco, and use of filter tips), and smoking behavior (frequency and depth of smoke inhalation).

#### Statistical analyses

Medians and 25th and 75th percentiles (interquartile range, IQR) of salivary cotinine concentration were computed according to the different strata of the potential modifiers. Median cotinine concentrations across the different variables were compared using non-parametric test for medians. Simple linear regression was used to derive the average increase in cotinine level (ng/ml) per one cigarette smoked, adjusting for the remaining variables. We analyzed the relation between number of cigarettes smoked in the previous 24 hours and salivary cotinine concentration using multiple linear regressions according to the strata of the potential modifiers of interest. Since the distributions of cotinine concentration and of the number of cigarettes were skewed, we first used log transformation, but the fit of the models did not improve. Following previous studies [9,18], we included a quadratic term for number of cigarettes to improve the models' fit. We assessed the improvement of fit between the adjusted and simple model with the *F *test statistic [19]. All models were tested for the applicability of conditions of linear regression (model specification, normality of errors, homoscedasticity, absence of multicollinearity, absence of outliers and lack of self-correlation). All analyses were performed using SPSS v13.0 (SPSS Inc., Chicago, IL).

## Results

Among the 211 current daily smokers (104 men and 107 women), the median age was 42.0 years (IQR 31.0-53.0). The sample was uniformly distributed across educational levels. The majority of participants (56%) were of normal weight and 19.4% had smoked more than 20 cigarettes in the last 24 hours. The median number of cigarettes smoked according to selected sociodemographic and smoking characteristics is shown in Table 1. The median number of cigarettes smoked in the last 24 hours was 15.0 (IQR 8.0-20.0), with significantly higher consumption in men compared to women (*p *< 0.05). Differences in cigarette consumption were also found by type of tobacco: smokers of black tobacco had smoked more cigarettes in the last 24 hours (median: 20 cigarettes) than smokers of blond tobacco (median: 12 cigarettes, p < 0.05).

**Table 1:** Median number of cigarettes smoked in the last 24 hours and interquartile ranges in adult daily smokers, according to individual characteristics, type of tobacco, and smoking characteristics.

	***n***	*median *(IQR*)	***p*-value^†^**
**Total**	211	15.0 (8.0, 20.0)	**-**
**Individual characteristics**			
*Sex*			0.002
Men	104	20.0 (10.0, 25.0)	
women	107	10.0 (6.0, 20.0)	
*Age (years)*			0.073
17-44	120	12.5 (8.3, 20.0)	
45-64	79	19.0 (10.0, 25.0)	
≥ 65	12	8.5 (4.8, 18.0)	
*Educational level*			0.162
Less than primary and primary	68	16.5 (10.0, 20.0)	
secondary	74	14.5 (8.8, 20.0)	
university	68	11.5 (6.0, 20.0)	
*Body mass index (kg/m*^2^)^‡^			0.705
underweight	4	10.0 (7.0, 17.5)	
normal	116	14.0 (8.0, 20.0)	
overweight	66	15.0 (8.0, 20.8)	
obese	21	20.0 (8.5, 30.0)	
**Type of tobacco**			
*Type of cigarettes*			0.040
regular	148	15.0 (8.5, 20.0)	
non-regular (light, ultralight, etc.)	63	10.0 (8.0, 20.0)	
Type of tobacco			0.003
blond	171	12.0 (8.0, 20.0)	
black	40	20.0 (10.5, 28.8)	
*Use of filter tip*			0.207
with filter	209	15.0 (8.0, 20.0)	
without filter	2	7.0 (4.0, 10.0)	
**Smoking characteristics**			
*Frequency of inhalation*			0.014
all the time	21	20.0 (10.5, 20.0)	
half the time	150	11.0 (7.0, 20.0)	
seldom	39	20.0 (12.0, 30.0)	
*Depth of inhalation*			0.739
light	22	16.0 (10.0, 22.5)	
moderate	78	12.0 (7.9, 20.0)	
deep	108	15.0 (8.0, 20.0)	

Barcelona (Spain), 2004-2005.

* *IQR*: interquartile range

† non-parametric test for medians

‡ underweight: <18.50 kg/m2; normal: 18.50-24.99 kg/m2; overweight: 25.00-29.99 kg/m2; obese: _30.00 kg/m2

Median cotinine concentrations by individual characteristics and smoking parameters are shown in Table 2. The overall median cotinine concentration was 146.5 ng/ml (IQR 86.8-220.5). Median cotinine concentration differed significantly by sex (172.6 ng/ml for men and 120.7 ng/ml for women, *p *< 0.001) regardless of the number of cigarettes smoked. Significant differences in cotinine concentrations were not found by age, educational level, or BMI (Table 2).

**Table 2:** Median cotinine concentrations (ng/ml) and interquartile ranges in adult daily smokers, according to individual characteristics, type of tobacco, and smoking characteristics.

	***n***	*median *(IQR*)	***p*-value^†^**
**Total**	211	146.5 (86.8, 220.5)	**-**
**Individual characteristics**			
*Sex*			
men	104	172.6 (115.3, 255.4)	< 0.001
women	107	120.7 (69.6, 208.8)	
*Age (years)*			
17-44	120	127.1 (82.8, 297.1)	0.090
45-64	79	171.1 (103.9, 239.4)	
≥ 65	12	135.8 (54.6, 215.7)	
*Educational level*			
less than primary and primary	68	173.9 (106.1, 295.8)	0.194
secondary	74	134.4 (86.5, 216.2)	
university	68	137.7 (67.4, 194.7)	
*Body mass index (kg/m*^2^)^‡^			
underweight	4	132.3 (72.4, 281.8)	0.151
normal	116	127.9 (85.1, 219.5)	
overweight	66	155.3 (88.7, 223.3)	
obese	21	166.6 (91.2, 224.2)	
**Type of tobacco**			
*Type of cigarettes*			
regular	148	149.5 (96.6, 229.8)	0.313
non-regular (light, ultralight, etc.)	63	128.4 (74.7, 214.8)	
Type of tobacco			
blond	171	137.0 (84.5, 219.6)	0.043
black	40	180.2 (128.6, 259.5)	
*Use of filter tip*			
with filter	209	146.9 (89.7, 220.5)	0.157
without filter	2	41.9 (12.7, 71.0)	
**Smoking characteristics**			
*Frequency of inhalation*			
all the time	21	166.6 (101.5, 207.1)	0.473
half the time	150	137.1 (83.1, 220.2)	
seldom	39	177.3 (108.6, 248.8)	
*Depth of inhalation*			
light	22	151.3 (80.6, 306.9)	0.957
moderate	78	142.8 (98.8, 220.2)	
deep	108	146.7 (84.8, 213.7)	

Barcelona (Spain), 2004-2005.

* *IQR*: interquartile range

† non-parametric test for medians

‡ underweight: <18.50 kg/m2; normal: 18.50-24.99 kg/m2; overweight: 25.00-29.99 kg/m2; obese: _30.00 kg/m2

As shown in Table 2, we found no statistically significant differences in the salivary cotinine concentration by type of cigarettes, use of filter, frequency, or depth of inhalation. Median cotinine concentration was higher among smokers of black tobacco (180.2 ng/ml) than among smokers of blond tobacco (137.0 ng/ml; *p *= 0.043). This association was confounded by the higher median consumption by smokers of black tobacco (Table 1), and by the predominance of men (70%) among users of black tobacco. There was no association between the type of tobacco smoked and cotinine concentration within strata of number of cigarettes smoked (1-9, 10-19, and = 20 cigarettes in the last 24 hours) or of sex (data not shown).

Coefficients (*β*) derived from simple linear regression estimate the average increase in cotinine concentration per one cigarette smoked during the previous 24 hours (Table 3). The increase in cotinine concentration per one cigarette smoked was 5.3 ng/ml in men and 7.7 ng/ml in women. No consistent trend was found in cotinine concentration by age; the greatest increase was observed in participants <45 years (*β *= 7.0; 95% CI, 5.2 - 8.8 ng/ml). Cotinine level increase per one cigarette smoked was greater among subjects who were at normal weight (8.2 ng/ml) and overweight (6.5 ng/ml) than in those who were obese (1.9 ng/ml). By type of tobacco, the greatest increase in cotinine concentration per one cigarette smoked was found in those smoking non-regular cigarettes (8.4 ng/ml) and in smokers of blond tobacco (6.9 ng/ml). Consistent trends were not found by use of filter tip, frequency, or depth of inhalation.

**Table 3:** Average increase in cotinine concentration (ng/ml) in adult daily smokers per one cigarette smoked in the previous 24 hours, according to individual characteristics, type of tobacco, and smoking characteristics. Barcelona (Spain), 2004-2005.

	***n***	β *	**95% CI^†^**	***R*^2^**
**Total**	211	6.4	5.2, 7.7	0.338
**Individual characteristics**				
*Sex*				
men	104	5.3	3.5, 7.2	0.244
women	107	7.7	5.9, 9.5	0.412
*Age (years)*				
17-44	120	7.0	5.2, 8.8	0.329
45-64	79	5.8	3.9, 7.8	0.329
≥ 65	12	6.1	-0.2, 12.5	0.316
*Educational level*				
less than primary and primary	68	5.4	2.9, 7.8	0.226
secondary	74	6.5	4.6, 8.4	0.392
university	68	7.5	5.4, 9.6	0.435
*Body mass index (kg/m*^2^)^‡^				
underweight	4	--^§^	--^§^	--^§^
normal	116	8.2	6.5, 9.9	0.439
overweight	66	6.5	4.3, 8.7	0.350
obese	21	1.9	-0.8, 4.8	0.102
**Type of tobacco**				
*Type of cigarettes*				
regular	148	5.8	4.4, 7.2	0.321
non-regular (light, ultralight, etc.)	63	8.4	5.7, 11.1	0.395
Type of tobacco				
blond	171	6.9	5.5, 8.3	0.356
black	40	5.1	2.1, 8.0	0.244
*Use of filter tip*				
with filter	209	6.3	5.1, 7.6	0.333
without filter	2	--^§^	--^§^	--^§^
**Smoking characteristics**				
*Frequency of inhalation*				
all the time	21	5.9	2.3, 9.5	0.384
half the time	150	8.4	6.8, 9.9	0.439
seldom	39	3.3	0.6, 6.0	0.147
*Depth of inhalation*				
light	22	7.9	2.8, 13.0	0.348
moderate	78	4.9	2.7, 7.0	0.217
deep	108	6.8	5.2, 8.3	0.416

Barcelona (Spain), 2004-2005.

* estimates from simple linear regression

† 95% confidence interval of _

‡ underweight: <18.50 kg/m2; normal: 18.50-24.99 kg/m2; overweight: 25.00-29.99

kg/m2; obese: _30.00 kg/m2

§not computed for insufficient observations

**Figure 1:**
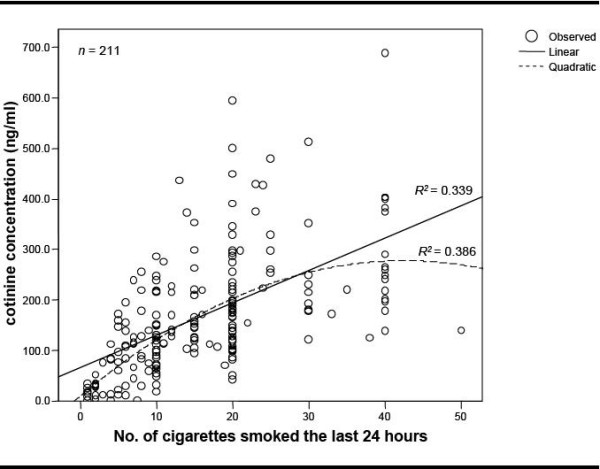
**The distribution of salivary cotinine concentration in relation to the number of cigarettes smoked during the 24 hours prior to saliva collection**.

The distribution of salivary cotinine concentration in relation to the number of cigarettes smoked during the 24 hours prior to saliva collection is shown in Fig 1. In the simple unadjusted linear model the number of cigarettes smoked in the last 24 hours was a predictor of cotinine concentrations (*R*^2 ^= 0.339; solid line). A significant improvement of the fit was obtained with a quadratic model, in which the number of cigarettes smoked accounted for almost 39% (adjusted *R*^2^) of the variance, and the exposure-response relationship leveled-off near 20 cigarettes (Fig. 1; dashed line). [see Additional file 1]

**Figure 2:**
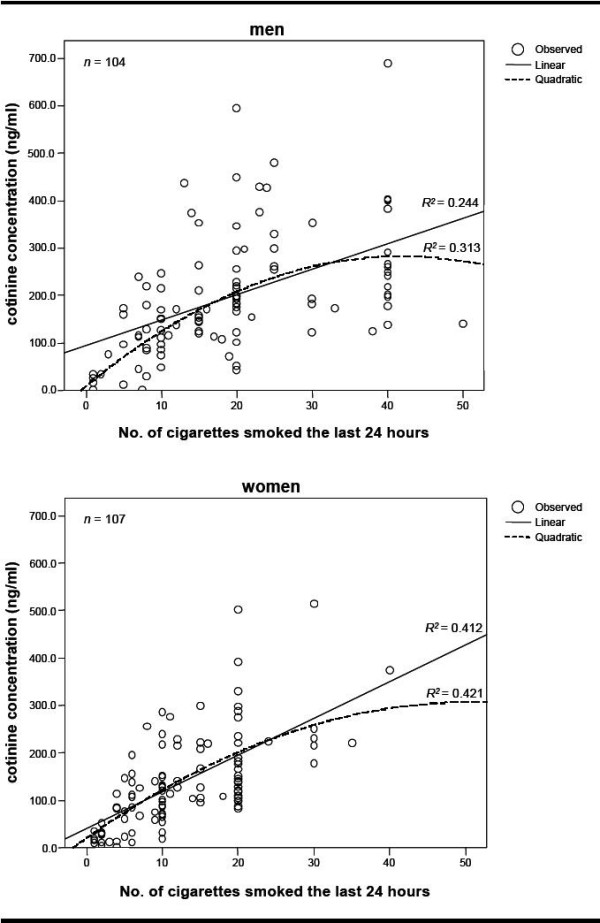
**The regression lines from linear and quadratic models for men and women**.

Due to differences found in cotinine concentrations between men and women, we analyzed these groups separately (Table 4). We observed an increment of 15.7 and 11.2 ng/ml in cotinine concentration per one cigarette smoked at a consumption of 1 cigarette per day in the quadratic model for men and for women, respectively. Further adjustment for age, educational level, and BMI in the quadratic model showed the best fit of the data with a similar increase in the cotinine concentration by cigarettes smoked (Table 4). Adjustment for other potential confounders identified in the bivariate analysis did not improve the model fit and hence these variables were not included in the final models (data not shown). Fig 2 shows the regression lines from linear and quadratic models for men and women. We verified the final models for error specification, normality, homoscedasticity, multicollinearity, outliers and self-correlation, and all diagnostics showed that the chosen models fulfilled the assumption [see Additional file 1]

**Table 4:** Average increase in cotinine concentration (ng/ml) in adult daily smokers per one cigarette smoked in the previous 24 hours by type of regression model.

**Estimation of model**	β *	**95% *CI***	***R*^2^**	***p*-value^†^**
**Men **(n = 103)				
simple linear model	5.3	3.5, 7.2	0.244	----
quadratic model	15.7	9.0, 22.3	0.313	< 0.05
quadratic model adjusted for covariates^†^	14.8	8.1, 21.5	0.353	< 0.05
**Women **(n = 104)				
simple linear model	7.7	5.9, 9.5	0.412	----
quadratic model	11.2	5.7, 16.7	0.421	0.188
quadratic model adjusted for covariates^‡^	11.3	5.8, 16.7	0.458	0.078

Barcelona (Spain), 2004-2005.

*β *for the simple variable "number of cigarettes smoked in previous 24 hours" (for one cigarette smoked)

*p*-value for the change of fit between the adjusted models and the simple linear model adjusted for age, educational level, and body mass index

## Discussion

This is the first study on smoking behavior in a Spanish adult population using both a questionnaire and a biomarker of tobacco exposure. Salivary cotinine concentration was associated with the number of cigarettes smoked in the last 24 hours. This relation was better explained with a quadratic function and in separate strata for men and women. The greater the number of cigarettes smoked, the greater the cotinine concentration in a linear scale up to 20 cigarettes per day, after which the association plateaus. A similar relation between salivary [6-9,20] or serum [21] cotinine concentrations and number of cigarettes smoked has been observed in other studies. Abrams et al. dichotomized tobacco consumption, and found that for smokers of less than 25 cigarettes per day, salivary cotinine concentration was highly correlated with tobacco consumption, while among heavier smokers the relation was not evident [22].

Other studies have found that cotinine concentration plateaus at different numbers of cigarettes: 25 cigarettes per day [23], 5 cigarettes per day [24], and 4 cigarettes per day in adolescent smokers [25]. The evidence suggests that cotinine concentration rises in a non-linear fashion with increasing number of cigarettes smoked, but the point where concentrations level off may vary across different populations. This finding suggests a difference in how people regulate their intake of nicotine to reach the desired dose [2], even for adolescents, who may be more susceptible to nicotine than adults and require only 4-5 cigarettes per day to satisfy their nicotine cravings [25].

We observed that cotinine concentrations differed by sex, regardless of the number of cigarettes smoked. Some studies reported similar findings of higher cotinine concentrations in men than in women [7,23,26,27], but other studies did not find differences by sex [6,28]. Association between urinary cotinine and cigarettes smoked according to sex was found in a study in the USA: urinary cotinine concentrations in men increased up to 34 cigarettes per day and then declined, while no flattening was observed in women [29]. The differences we observed by sex could reflect not only differences in tobacco consumption by sex, i.e., men usually smoke more cigarettes than women, but also a difference in the metabolism of nicotine between men and women [30,31].

Our data showed a higher cotinine concentration in smokers of black tobacco that did not persist with control for the number of cigarettes smoked. Whereas uptake of carcinogens is higher among black tobacco smokers [32-34], differences in nicotine uptake by type of tobacco smoked have not been reported [35]. In our study, use of filter, frequency, and depth of inhalation were not related to cotinine concentrations. An explanation of these results could be that smokers tend to maintain the same intake level of nicotine by drawing in more smoke per cigarette when they try to smoke fewer cigarettes. Benowitz et al. reported that among people who reduced from 37 to 5 cigarettes per day on average, the intake of tobacco toxins per cigarette increased roughly threefold [36]. This could also explain how cotinine concentrations level-off in smokers of more than 20 cigarettes per day, when a certain intake of nicotine is achieved [8,9].

The role of age in cotinine concentrations is still not clear, since our results, as well as previous studies [7,37], indicated no association between cotinine concentrations and age, while others have found a significant association [6,18,29,38]. Some studies have modeled the relation between cotinine concentrations and cigarette consumption by taking into account several of these variables. The fit of the multivariate model improved once age, BMI, educational level, and a quadratic term for cotinine were included.

### Study limitations and strengths

To our knowledge, this is one of the few studies in which information about tobacco exposure was obtained in a representative random sample of the general population with a simultaneous use of a questionnaire and a biological marker. In the USA, the National Health and Nutrition Examination Survey (NHANES) provides population-based national estimates of smoking prevalence using both standard questionnaire and serum cotinine concentration [39]. Most previous studies were based on selective samples from existing observational studies [6,8,18,25,37] or smoking cessation trials [21,22,29]. Other factors affecting validity also need to be considered. Although the use of self-reported data from questionnaires could be a source of bias, self-reports on smoking are accurate and have acceptable validity [40,41]. Cotinine is a specific biomarker of tobacco exposure [1,2], and the laboratory methods are highly sensitive [17].

Some potential limitations deserve consideration. We found that the model fit could be affected by the measurement scale of the number of cigarettes. Smokers tend to round up the number of cigarettes smoked, particularly heavy smokers [42], and hence some information bias due to digit preference cannot be disregarded. While some loss of representativeness due to non-response might also be possible, the sample did not differ by sex, age, and district of residence from the Barcelona population. Moreover, the prevalence of smokers in the sample (28.6% of men and 18.2% of women) was similar to that derived from the 2006 Health Interview Survey of Barcelona (27.3% of men and 20.6% of women) [43]. The participation rate was almost complete because the study design allowed replacement of non-respondents by subjects in the same strata of sex, age, and district of residence.

## Conclusion

Cotinine concentration differed by sex and increased up to consumption of 20 cigarettes per day and then flattened at higher levels of smoking. Further investigation may help to better understand the relationship between number of cigarettes smoked, age, sex, weight, subjects' levels nicotine or cotinine concentrations, and the degree of nicotine dependence that may have implications in smoking cessation.

## Competing interests

The authors declare that they have no competing interests.

## Authors' contributions

The DCOT investigators designed the study. JT, AS and MF co-ordinated participant recruitment, collected data and were responsible for data management. JAP was responsible for cotinine analyses. MF, JMMS, and EF performed statistical analyses. All authors participated in the interpretation of results. MF drafted the manuscript, and all authors contributed to the critical review and revision of the manuscript. All authors approved the final version of the manuscript. EF is the guarantor.

## Pre-publication history

The pre-publication history for this paper can be accessed here:



## Supplementary Material

Additional file 1 **Supplemental material**. Salivary cotinine concentration (ng/ml) in adult daily smokers in relation to the number of cigarettes smoked in the last 24 hours. Barcelona (Spain), 2004-2005, and Salivary cotinine concentration (ng/ml) in adult daily smokers in relation to the number of cigarettes smoked in the last 24 hours, in separate strata for men and women. Barcelona (Spain), 2004-2005. Click here for file
