# Supplementary material for: Salivary cotinine concentrations in daily smokers in Barcelona, Spain: a cross-sectional study
Source: BMC Public Health. 2009 Sep 3;9:320. doi: 10.1186/1471-2458-9-320 (PMC2749042; doi:10.1186/1471-2458-9-320)
Supplement: Additional file 1 — Supplemental material. Salivary cotinine concentration (ng/ml) in adult daily smokers in relation to the number of cigarettes smoked in the last 24 hours. Barcelona (Spain), 2004-2005, and Salivary cotinine concentration (ng/ml) in adult daily smokers in relation to the number of cigarettes smoked in the last 24 hours, in separate strata for men and women. Barcelona (Spain), 2004-2005. [file 1471-2458-9-320-S1.pdf]

Determinantes de los niveles de cotinina en saliva en una muestra representativa de la población general.

Institut Català d'Oncologia

CSB Consorci Sanitari de Barcelona
Agència de Salut Pública

IMAS
Institut Municipal d'Investigació Mèdica. IMIM

Generalitat de Catalunya
Departament de Sanitat i Seguretat Social

JOHNS HOPKINS BLOOMBERG SCHOOL of PUBLIC HEALTH

DCOT

Estudi dels determinants de la cotinina
Estudio de los determinantes de la cotinina

Servei de Prevenció. ICO
Av. Gran Via s/n, km 2,7
08907 L'Hospitalet. Barcelona
Telf. 93 260 77 88

Código	
Hora de inicio	
Fecha de realización	
Entrevistador	
Hora de finalización	
Tipo cuestionario	
Idioma cuestionario	Castellano

HOJA SEPARABLE

Etiqueta:

Nombre y Apellidos
Dirección y Teléfono
Código

CONSENTIMIENTO PARA LA ENTREVISTA
Y LA OBTENCIÓN DE MUESTRAS DE SALIVA EN EL ESTUDIO
DETERMINANTES DE LOS NIVELES DE COTININA EN SALIVA EN UNA MUESTRA REPRESENTATIVA DE LA POBLACIÓN GENERAL

Yo, Sr./Sra.____________________________________ de ______ de edad y con DNI ___________

DECLARO

Que he sido informado/da por el Sr./Sra. ____ colaborador/a del Estudio "Determinantes de los niveles de cotinina en saliva en una muestra representativa de la población general", que:

l'Institut Català d'Oncologia esta llevando a cabo un estudio sobre el consumo de tabaco y la exposición al tabaco de la población de Barcelona,

se ha solicitado mi participación voluntaria en este estudio, que supone responder un cuestionario confidencial sobre consumo de tabaco y proporcionar 8ml. de mi saliva,

la información recogida en este estudio es confidencial y sólo los investigadores conocen la identidad de los que participan. La saliva se utilizará para la determinación de cotinina u otros marcadores de exposición al tabaco relacionados con la finalidad del estudio,

la saliva no utilizada en esta primera fase del estudio se congelará y podrá ser utilizada para futuras determinaciones relacionadas con las finalidades del estudio,

la publicación de los resultados no revelará en ningún caso la identidad de las personas participantes,

he sido informado/da de forma clara y comprensible de la finalidad, limitaciones y beneficios de este estudio, y que me han contestado a todas las preguntas que he hecho y dudas que he mostrado al respecto.

También he sido informado/da de que en cualquier momento puedo retirarme del estudio y anular mi consentimiento.

Por estas razones, **ACCEDO** a contestar el cuestionario, **AUTORIZO** la recogida de saliva y doy mi **CONSENTIMIENTO INFORMADO** para que esta información y la muestra de saliva sea utilizada por los investigadores en el estudio que están llevando a cabo con tal de mejorar y ampliar los conocimientos sobre el tabaquismo.

Firma del participante	Firma del entrevistador/a
Nombre y apellidos:	Nombre y apellidos:
DNI:	DNI:

Barcelona a, ___ / _____________ / ______

Tal y como ya le he comentado, a continuación le haré unas preguntas sobre su salud general, sus estilos de vida y algunos datos sociodemográficos. Le pido, por favor, que me conteste con toda sinceridad y libertad, ya que no hay respuestas correctas ni incorrectas. ¿Empezamos? Sí / No (Marcar con un círculo)

P0A. ¿Me podría decir usted su fecha de nacimiento?

	día	mes	año	NS/NC
Fecha de nacimiento				

P0B. ¿Y su país de nacimiento?

SALUD EN GENERAL

A continuación, le haré preguntas referentes a su estado de salud. En algunos casos me habrá de contestar directamente y en otros le leeré las respuestas. Por favor, conteste cuando le haya leído todas las opciones

P1. ¿Cómo diría usted que es su salud en general? (Leer las categorías)

Excelente	
Muy Buena	
Buena	
Regular	
Mala	
NS/NC	

MORBILIDAD

P2. ¿Me podría decir si sufre, o si su médico le ha dicho que sufre alguno de los trastornos crónicos que ahora le leeré?

	Sí	No	NS/NC
Presión alta			
Enfermedades del corazón			
Varices en las piernas			
Artrosis, artritis, reumatismo			
Dolor crónico de espalda			
Alergias crónicas			
Asma			
Bronquitis crónica			
Diabetes			
Úlcera de estómago o duodeno			
Molestias urinarias			
Problemas de próstata (No preguntar si es mujer)			
Colesterol elevado			
Cataratas			
Problemas crónicos de piel			
Estreñimiento crónico			
Depresión/ ansiedad u otros trastornos mentales			
Embolia			
Migraña			
Mala circulación			
Hemorroides			
Problemas de visión			
Otros (Especificar): ✍			

VISITAS MÉDICAS

P3. Durante estos últimos 15 días, ¿usted ha sido visitado por un profesional sanitario por motivo de su salud?

Sí		
No		→ Pasar al siguiente apartado
NS/NC		

P4. ¿A cuál de los siguientes profesionales sanitarios visitó?

	Sí	No	NS/NC
Médico de medicina general			
Pediatra (No preguntar si es >14 años)			
Oculista			
Dentista			
Médico especialista (cardiólogo, psiquiatra, neurólogo, ginecólogo, etc.)			
Fisioterapeuta			
Enfermera			
Psicólogo			
Homeópata			
Naturista			
Acupuntor			
Otros profesionales: ✍			

ESTILOS DE VIDA

MENORES DE 12 AÑOS PASAR A LA PREGUNTA 51

TABACO

A continuación le haré unas preguntas sobre su consumo de tabaco

SÓLO PARA LOS ADOLESCENTES (12 - 16 años)

P5. ¿Con cuál de las siguientes afirmaciones te identificas más? (Marcar sólo una respuesta)

Fumo al menos un cigarrillo al día		→ Pasar a la pregunta 7
No fumo diariamente, pero sí al menos un cigarrillo a la semana		→ Pasar a la pregunta 36
No fumo semanalmente, pero sí al menos una vez al mes		→ Pasar a la pregunta 36
Fumo menos de una vez al mes		→ Pasar a la pregunta 36
Fumo alguna vez de tanto en tanto		→ Pasar a la pregunta 36
He dejado de fumar, después de haber fumado al menos una vez a la semana		→ Pasar a la pregunta 45
He dejado de fumar, siempre fumé menos de una vez a la semana		→ Pasar a la pregunta 45
Fumé alguna vez, pero ya no he fumado nunca más		→ Pasar a la pregunta 45
Nunca he fumado, ni lo he probado		→ Pasar a la pregunta 51
NS/NC		

PARA TODOS LOS ADULTOS (>16 años)

P6. De las siguientes afirmaciones, indique cuál describe mejor su comportamiento respecto al tabaco

Actualmente fumo cada día (al menos 1 cig/día)		
Actualmente fumo ocasionalmente		→ Pasar a la pregunta 36
Ahora no fumo, pero antes fumaba cada día		→ Pasar a la pregunta 45
Ahora no fumo, pero antes fumaba ocasionalmente (mínimo 1 cig/día)		→ Pasar a la pregunta 45
No he fumado nunca		→ Pasar a la pregunta 51
NS/NC		

SÓLO PARA LOS FUMADORES ACTUALES (DIARIOS + OCASIONALES)

P7. ¿A qué edad comenzó a fumar regularmente?

Edad	
NS/NC	

P8. ¿Qué tipo de tabaco consume habitualmente? (Puede marcar más de una respuesta)

Cigarrillos	
Puros o cigarros	
Puritos (Farias, Reig, Señoritas, Caliqueños,…)	
Pipa	
Tabaco de liar	
Otros: (Tabaco de mascar, porros, rape)	
NS/NC	

P9. ¿Cuántos _________ ha fumado en las últimas 24 horas? (Puede marcar más de una respuesta)

Cigarrillos	
Puros o cigarros	
Puritos (Farias, Reig, Señoritas, Caliqueños,…)	
Pipa	
Tabaco de liar	
Otros: (Tabaco de mascar, porros, rape)	
NS/NC	

P10. ¿Cuántos __________ en total ha fumado en las últimas 48 horas? (Puede marcar más de una respuesta)

Cigarrillos	
Puros o cigarros	
Puritos (Farias, Reig, Señoritas, Caliqueños,...)	
Pipa	
Tabaco de liar	
Otros: (Tabaco de mascar, porros, rape)	
NS/NC	

P11. En promedio, ¿cuántos _________ fuma normalmente al día?

	Entre semana o día laborable	Fin de semana o tiempo libre
Cigarrillos		
Puros o cigarros		
Puritos (Farias, Reig, Señoritas, Caliqueños,…)		
Pipa		
Tabaco de liar		
Otros: (Tabaco de mascar, porros, rape)		
NS/NC		

P12. De los __ (tipo de tabaco más consumido) que ha dicho que fuma normalmente, ¿cuántos fuma…?

	Entre semana o día laborable		Fin de semana o tiempo libre	
		NS/NC		NS/NC
En casa				
En el trabajo			-	-
En el transporte				
Otros lugares				

P13. Habitualmente cuando enciende un cigarrillo…

Estoy continuamente haciendo caladas hasta que se consume	
Hago caladas de manera regular pero no continuamente	
Hago pocas caladas y el cigarrillo se quema solo	
NS/NC	

P14. ¿Con qué profundidad inhala el humo de los cigarrillos?

Superficial, hasta a la boca	
Intermedia, hasta el cuello	
Profunda, hasta los pulmones	
NS/NC	

P15. ¿Qué marca o marcas fuma habitualmente? (La que fuma normalmente, la más frecuente)

	Marca
La más habitual	
La segunda más habitual	
NS/NC	

P16. ¿Qué marca o marcas, ha fumado las últimas 48 horas?

	Marca
La más fumada	
La segunda más fumada	
NS/NC	

P17. ¿Qué clase de cigarrillos fuma usted habitualmente?

Normal	
“Light”, “Ultralight”, Bajo en nicotina, mentolados,…	
NS/NC	

P18. ¿Qué tipo de cigarrillos fuma usted habitualmente?

Rubio	
Negro	
NS/NC	

P19. ¿Qué tipo de cigarrillos fuma usted habitualmente?

Con filtro	
Sin filtro	
NS/NC	

P20. ¿Dónde acostumbra a comprar el tabaco?

	Sí	No	NS/NC
Estanco			
Bar/ Cafetería/ Quiosco			
Máquina expendedora			
En la calle/ Metro (vendedores ambulantes)			

P21 Durante la última semana, ¿ha comprado…?

	Sí	No	NS/NC
Paquetes de 20 cigarrillos			
Paquetes de 10 cigarrillos			
Cartones (10 paquetes)			
Cigarrillos a granel			

P22. ¿Cuántos centímetros deja hasta el filtro del cigarrillo, antes de apagarlo? ((Enseñar cigarrillo muestra)

Cm	
NS/NC	

P23. ¿Cuánto tiempo pasa hasta fumar el primer cigarrillo después de levantarse?

< 5 minutos	
5 – 15 minutos	
16 – 30 minutos	
31 minutos – 1 hora	
1 – 2 horas	
Más de 2 horas	
NS/NC	

P24. ¿Le cuesta no fumar en lugares donde está prohibido hacerlo (cine, autobús,…)?

Sí	
No	
NS/NC	

P25. ¿A qué cigarrillo le cuesta más renunciar?

Al de primera hora de la mañana	
Otros	
NS/NC	

P26. ¿Fuma más durante las primeras horas de la mañana que durante el resto del día?

Sí	
No	
NS/NC	

P27. Si está tan enfermo que se queda la mayor parte del día en la cama, ¿fuma?

Sí	
No	
NS/NC	

P28. ¿El médico le ha aconsejado alguna vez dejar de fumar?

Sí	
No	
No ha ido nunca al médico	
NS/NC	

Sí → ¿Público o privado? Público ☐ Privado ☐

P29. ¿Está considerando, seriamente, reducir el número de cigarrillos que fuma habitualmente?

Sí	
No	
NS/NC	

P30. En el último año, ¿ha estado al menos un día sin fumar? (los días en que ha estado enfermo no cuentan)

Sí	
No	
NS/NC	

P31. En la actualidad, ¿está dejando de fumar?

Sí	
No	
NS/NC	

No → Pasar a la pregunta 33

P32. ¿Qué tratamiento está utilizando?

	Sí	No	NS/NC
Chicles de nicotina			
Parches de nicotina			
Hierbas			
Terapia de grupo			
Bupropion			
Otros. Especificar:			

PASAR A LA PREGUNTA 34

P33. Está considerando seriamente la posibilidad de…

Dejar de fumar durante la próxima semana	
Dejar de fumar durante el próximo mes	
Dejar de fumar durante los próximos 3 meses	
Dejar de fumar durante los próximos 6 meses	
Dejar de fumar durante los próximos 12 meses	
Dejar de fumar pero no durante los próximos 12 meses	
No intentaré dejar de fumar	
NS/NC	

P34. ¿Ha intentado dejar de fumar en los últimos 12 meses? (Marcar con un círculo el 0 si no lo ha intentado)

	Nº de intentos
Sí	
No	0
NS/NC	

P35. ¿Cuál ha sido el período más largo de tiempo que ha estado sin fumar en los últimos 5 años? (los días en que ha estado enfermo no cuentan)

No lo he dejado nunca	
Menos de un día	
Un día	
Hasta una semana	
Hasta 4 semanas	
Hasta 3 meses	
Hasta 6 meses	
Hasta 12 meses	
Más de 12 meses	
NS/NC	

PASAR A LA PREGUNTA 51

SÓLO PARA LOS FUMADORES OCASIONALES

P36. ¿A qué edad comenzó a fumar?

Edad	
NS/NC	

P37. ¿Qué tipo de tabaco consume habitualmente? (Puede marcar más de una respuesta)

Cigarrillos	
Puros o cigarros	
Puritos (Farias, Reig, Señoritas, Caliqueños….)	
Pipa	
Tabaco de liar	
Otros: (Tabaco de mascar, porros, rape)	
NS/NC	

P38 ¿Cuántos _________ ha fumado en las últimas 24 horas? (Puede marcar más de una respuesta)

Cigarrillos	
Puros o cigarros	
Puritos (Farias, Reig, Señoritas, Caliqueños….)	
Pipa	
Tabaco de liar	
Otros: (Tabaco de mascar, porros, rape)	
NS/NC	

P39. ¿Cuántos __________ en total ha fumado en las últimas 48 horas? (Puede marcar más de una respuesta)

Cigarrillos	
Puros o cigarros	
Puritos (Farias, Reig, Señoritas, Caliqueños….)	
Pipa	
Tabaco de liar	
Otros: (Tabaco de mascar, porros, rape)	
NS/NC	

P40. ¿Dónde acostumbra a comprar el tabaco?

	Sí	No	NS/NC
Estanco			
Bar/ Cafetería/ Quiosco			
Máquina expendedora			
En la calle/ Metro (vendedores ambulantes)			

P41. Durante la última semana, ¿ha comprado…?

	Sí	No	NS/NC
Paquetes de 20 cigarrillos			
Paquetes de 10 cigarrillos			
Cartones (10 paquetes)			
Cigarrillos a granel			

P42. ¿El médico le ha aconsejado alguna vez dejar de fumar?

Sí	
No	
No ha ido nunca al médico	
NS/NC	

→ ¿Público o privado? Público ☐ Privado ☐

P43. Está considerando seriamente la posibilidad de…

Dejar de fumar durante la próxima semana	
Dejar de fumar durante el próximo mes	
Dejar de fumar durante los próximos 3 meses	
Dejar de fumar durante los próximos 6 meses	
Dejar de fumar durante los próximos 12 meses	
Dejar de fumar pero no durante los próximos 12 meses	
No intentaré dejar de fumar	
NS/NC	

P44. ¿Ha intentado dejar de fumar en los últimos 12 meses? (Marcar con un círculo el 0 si no lo ha intentado)

	Nº de intentos
Sí	
No	0
NS/NC	

PASAR A LA PREGUNTA 51

SÓLO PARA LOS EXFUMADORES (DIARIOS+OCASIONALES)

P45. ¿A qué edad empezó a fumar regularmente?

Edad	
NS/NC	

P46. ¿Qué cantidad y qué tipo de tabaco fumaba Ud. habitualmente cada día?

	Cantidad
Cigarrillos	
Puros o cigarros	
Puritos (Farias, Reig, Señoritas, Caliqueños…)	
Pipa	
Tabaco de liar	
Otros (Tabaco de masticar, porros, rapé)	
NS/NC	

P47. ¿A qué edad dejó de fumar?

Edad	
NS/NC	

P48. ¿Qué marca o marcas fumaba habitualmente? …

	Marca
La más habitual	
La segunda más habitual	
NS/NC	

P49. Cuando era fumador, ¿el médico le había aconsejado alguna vez dejar de fumar?

Sí	
No	
No ha ido nunca al médico	
NS/NC	

→ ¿Público o privado? Público ☐ Privado ☐

P50. ¿Cuáles fueron los dos principales motivos para dejar de fumar? (Marcar sólo 2 motivos y el orden en que los dice)

Me lo aconsejó el médico u otro profesional sanitario	
Tenía molestias a causa del tabaco	
Aumentó mi grado de preocupación por los efectos nocivos del tabaco (riesgo para la salud)	
Noté que disminuía mi rendimiento psíquico y/o físico, en general	
Lo decidí solo por propia voluntad	
Por estar embarazada o planificando el embarazo	
Otros motivos (Especificar): ✍	
NS/NC	

TODOS LOS SUJETOS

EXPOSICIÓN PASIVA AL HUMO AMBIENTAL DEL TABACO

DOMICILIO

Ahora le haré unas preguntas sobre la exposición que tiene al humo ambiental del tabaco en diferentes ambientes.

P51. Actualmente, ¿Cuántas personas fuman habitualmente dentro de su casa? ☐ (Nº de personas)

	Número de cigarrillos	Parentesco
Persona 1		
Persona 2		
Persona 3		
Persona 4		
Persona 5		
Persona 6		
Nadie		-
NS/NC		

(Persona 1: Es el propio entrevistado en caso de ser fumador)

P52. Durante la semana pasada, ¿cuántos cigarrillos se han fumado al día en su presencia en su casa?

	Entre semana o día laborable	Fin de semana o tiempo libre
Nº de cigarrillos		
NS/NC		

P53. ¿Qué situación describe mejor las "normas" sobre el fumar dentro de su casa?

Nadie puede fumar	
Sólo se puede fumar en algunos lugares dentro de casa. Especificar: ✍	
Se puede fumar en todas partes (No hay normas)	
NS/NC	

P54. ¿Le llega olor de tabaco a su casa desde el exterior?

		No
Origen 1	Especificar: ✍	
Origen 2	Especificar: ✍	
Origen 3	Especificar: ✍	
NS/NC		

MENORES DE 16 AÑOS PASAR A LA PREGUNTA 66

LABORAL

P55. ¿Trabaja usted fuera de casa?

Sí	
No	→ Pasar a la pregunta 63
NS/NC	

P56. En su lugar de trabajo ¿hay algún tipo de regulación respecto al consumo de tabaco?

Sí		¿Desde cuándo? Hace menos de un año ☐ / Hace un año o más ☐
No		→ Pasar a la pregunta 58
NS/NC		→ Pasar a la pregunta 58

P57. ¿Las personas respetan esta regulación?

Sí	
No	
NS/NC	

P58. En su lugar de trabajo ¿existen espacios específicos para fumadores?

Sí	
No	
NS/NC	

P59. ¿Tiene usted algún compañero/a que fume cerca suyo en el trabajo? (Que le llegue el humo a su lugar de trabajo)

Sí		Especificar cuántas personas: ____ ✍
No		→ Pasar a la pregunta 63
NS/NC		

P60. ¿Cuántas horas cree que está expuesto al humo ambiental del tabaco durante su jornada laboral?

Nº de horas	
NS/NC	

P61. Respecto al humo del tabaco, ¿cómo describiría el ambiente de su lugar de trabajo?

Muy cargado	
Poco cargado	
Nada cargado	
NS/NC	

SÓLO PARA FUMADORES ACTUALES CON REGULACIÓN EN SU LUGAR DE TRABAJO

P62. ¿Cree que fuma menos cigarrillos por día debido a la regulación existente sobre consumo de tabaco en su lugar de trabajo?

Sí	
No	
NS/NC	

P63. ¿Es usted estudiante universitario?

Sí	
No	
NS/NC	

No → Pasar a la pregunta 68

SÓLO PARA ESTUDIANTES UNIVERSITARIOS

P64. ¿En cuáles de estos espacios de tu facultad hay señalización sobre consumo de tabaco? ¿Se cumple?

	Señalización			¿Se fuma?		
	Sí	No	NS/NC	Sí	No	NS/NC
Aula						
Pasillo / Vestíbulo						
Bar / Cafetería						
Lavabo						
Biblioteca						
Sala de estudio						
Copistería						

P65. ¿Cuántas horas al día como promedio cree que está expuesto al humo ambiental del tabaco durante su estancia en la facultad?

Nº de horas	
NS/NC	

PASAR A LA PREGUNTA 68

SÓLO PARA ESCOLARES (Jardín de Infancia, Parvulario, Primaria, ESO, Bachillerato o ciclos formativos)

P66. Durante la semana pasada, ¿cuántos cigarrillos se han fumado en tu presencia en la escuela?

	Profesores	Compañeros
Número de cigarrillos		
NS/NC		

P67. Entre los profesores de tu escuela…

Todos o casi todos fuman	
Hay fumadores y no fumadores, más o menos por igual	
La mayoría no fuman, pero hay alguno que sí	
Nadie fuma	
NS/NC	

TODOS LOS ENTREVISTADOS

TRANSPORTES

P68. Durante la semana pasada, ¿ha ido en algún transporte donde se haya fumado?

	Público (metro, autobús, tren, taxi)	Coche particular (propio o de otros)
No		
Menos de 30 minutos/ día		
Entre 30 y 60 minutos/ día		
Más de 60 minutos/ día		
No ha utilizado transporte		
NS/NC		

TIEMPO LIBRE

P69. ¿Cuánto tiempo de «promedio» acostumbra a estar en ambientes con humo de tabaco fuera de casa y del trabajo?

	Entre semana o día laborable	Fin de semana o tiempo libre
Nada		
Menos de 1 hora		
De 1 a 4 horas		
Más de 4 horas		
NS/NC		

P70. ¿Dónde cree usted que ha estado expuesto durante la última semana al humo ambiental del tabaco?

	Nada	Poco	Mucho	No ha ido	NS/NC
Bar/Cafetería/Granja					
Restaurante					
Discoteca/Pubs/Bar musical					
Grandes almacenes o superficies comerciales					
Espacios culturales (teatro, cine)					
Otros (Especificar): ✍					

P71. Entre los amigos con los que sale más…

Todos o casi todos fuman	
Hay fumadores y no fumadores, más o menos por igual	
La mayoría no fuman, pero hay alguno que sí	
Nadie fuma	
NS/NC	

P72. Durante un día normal de la semana pasada, enumere de más (1) a menos (4) el lugar donde se ha sentido más expuesto al humo ambiental del tabaco. (Marcar como "Nada " en caso de no haber estado expuesto en alguno de los ambientes)

	Entre semana o día laborable	Fin de semana o tiempo libre
En casa		
En el trabajo/escuela		-
En su tiempo libre		
En los medios de transporte		
NS/NC		

PERCEPCIÓN DE RIESGO

P73. Responda a las siguientes afirmaciones que ahora le leeré según su grado de acuerdo: totalmente de acuerdo, …, totalmente en desacuerdo. (Se ha de forzar a que la gente se decante hacia a un lado o el otro). (Leer en las 3 primeras frases todas las alternativas: "usted está totalmente de acuerdo, de acuerdo, ni de acuerdo ni en desacuerdo, en desacuerdo, totalmente en desacuerdo…")

	Totalmente de acuerdo	De acuerdo	Ni de acuerdo ni en desacuerdo	En desacuerdo	Totalmente en desacuerdo	NS/NC
El humo ambiental del tabaco es molesto para mí						
Respirar humo del cigarrillo de otro es nocivo						
Fumar debería estar prohibido en todos los espacios públicos, incluidos bares y restaurantes						
El humo de tabaco es peligroso para los adultos						
El humo de tabaco es peligroso para los niños						
El humo de tabaco es peligroso para los no fumadores						

P74. ¿En los últimos 12 meses, dejó de ir a algún lugar público o privado por que sabía que estaría muy expuesto al humo ambiental del tabaco?

Sí	
No	
NS/NC	

P75. ¿Alguna vez ha dejado de ir a algún lugar por que estaba prohibido fumar?

Sí	
No	
NS/NC	

A continuación le preguntaré sobre la actividad física que realiza y sobre su consumo de bebidas alcohólicas

ACTIVIDAD FÍSICA

P76. De las siguientes posibilidades que ahora le leeré, indique qué opción describe mejor la actividad que realiza habitualmente en el trabajo, en el domicilio o en su lugar de estudios (excluye la actividad deportiva y de ocio):

Está sentado durante la mayor parte de la jornada	
Está de pie la mayor parte de la jornada sin desplazarse	
Su actividad no requiere un esfuerzo físico importante, pero se desplaza a pie frecuentemente	
Realiza una actividad que requiere esfuerzo físico importante	
Otros (Especificar): ✍	
NS/NC	

P77. Durante los últimos 15 días, ¿ha aprovechado alguna vez su tiempo libre para caminar o pasear?

Sí		
No		→ Pasar a la pregunta 80
NS/NC		

P78. ¿Cuántas veces ha caminado durante los últimos 15 días?

Veces	
NS/NC	

P79. ¿Cuántos minutos de promedio, aproximadamente, ha estado caminando cada vez?

Minutos por vez	
NS/NC	

P80. ¿Durante los últimos 15 días ha realizado algún tipo de actividad física o deportiva en su tiempo libre (como ahora yoga, footing, fútbol, esquí, caza, pesca, jardinería, danza, etc.)?

Sí		
No		→ Pasar a la pregunta 84
NS/NC		

P81. ¿Cuál o cuáles actividades físicas o deportivas ha realizado durante los últimos 15 días? (Encuestador: anotar la respuesta en la tabla siguiente)

P82. ¿Cuántas veces ha practicado esta actividad (o cada una de estas actividades) a lo largo de los últimos 15 días? (Encuestador: anotar la respuesta en la tabla siguiente)

P83. ¿Durante cuántos minutos aproximadamente ha practicado esta actividad (o cada una de estas actividades) cada vez? (Encuestador: anotar la respuesta en la tabla siguiente)

Actividades	Veces	Minutos por vez
1.		
2.		
3.		
4.		
5.		
NS/NC		

ALCOHOL

P84. Durante el último día entre lunes y jueves, ¿cuántos vasos, copas o consumiciones tomó de…?

	Nº de consumiciones
Cerveza	
Vino, cava y similares	
Coñac, "carajillos", vermut, licores y similares	
Whisky, ginebra, vodka, ron, aguardiente, combinados o similares	
NS/NC	

P85. ¿Y durante el último fin de semana (viernes + sábado + domingo)?

	Nº de consumiciones
Cerveza	
Vino, cava y similares	
Coñac, "carajillos", vermut, licores y similares	
Whisky, ginebra, vodka, ron, aguardiente, combinados o similares	
NS/NC	

DATOS SOCIO-DEMOGRÁFICOS

Para acabar, le preguntaré algunos datos personales y profesionales

P86. ¿Cuál es su estado civil?

Soltero/a	
Casado/da – Vive en pareja	
Viudo/a	
Separado/a	
Divorciado/a	
NS/NC	

P87. ¿Cuántas personas en total viven con usted de manera habitual en casa, incluyéndolo a usted?
(Marcar con un círculo la edad del entrevistado)

|__|__| personas Edades: |__|__|__| |__|__|__| |__|__|__| |__|__|__| |__|__|__|

P88. ¿Cuál es el máximo nivel de estudios que ha alcanzado usted? (No leer las alternativas de respuesta)

No sabe leer ni escribir	
No ha cursado estudios, pero sabe leer y escribir	
Estudios primarios incompletos (6º de EGB, ingreso o similar)	
Estudios primarios completos (EGB, grad. escolar, bachillerato elem., plan de antes BUP, FP1, “las 4 reglas”)	
Educación secundaria obligatoria (E.S.O)	
Estudios secundarios: BUP, COU, PREU, bachillerato plan nuevo	
FPII y ciclos formativos/ Fondos de garantía social	
Estudios universitarios de grado medio (diplomados)	
Estudios universitarios de grado superior (licenciados)	
Educación especial	
Otra posibilidad. Especificar: ✍	
NS/NC	

P89. ¿Cuál es su situación laboral actual?

Trabajaja		→ Pasar a la pregunta 91
Parado		
Incapacitado/a o invalidez permanente		
Jubilado/a		
Ama de casa		
Estudiante		
Otras posibilidades: ✍		
NS/NC		

P90. A pesar de que usted no trabaje en la actualidad, ¿me podría decir si trabajó anteriormente?

Sí		
No		→ Pasar a la pregunta 94
NS/NC		

PARA TODOS LOS QUE TRABAJAN O HAN TRABAJADO ANTERIORMENTE

P91. En su trabajo o en su último trabajo, ¿qué trabajo concreto hace (o hacía)? (Recoger el máximo de información)

____

P92. ¿Cuál es la actividad principal de la empresa donde trabaja o trabajaba? (Recoger el máximo de información)

____

P93. ¿Cuál es o cuál era su situación laboral en esa ocupación?

Asalariado	
Trabajador por cuenta propia, sin asalariados	
Trabajador por cuenta propia, con 10 o más asalariados	
Trabajador por cuenta propia, con menos de 10 asalariados	
Gerente de una empresa con 10 o más asalariados	
Gerente de una empresa con menos de 10 asalariados	
Capataz, Supervisor o encargado	
Otros (Becarios,…)	
NS/NC	

PASAR A LA PREGUNTA P99

PARA TODOS LOS ENTREVISTADOS QUE NO HAN TRABAJADO NUNCA

P94. ¿Cuál la situación laboral de la persona principal? (Persona principal, la que aporta o ha aportado más recursos económicos a la familia)

Trabaja	
Parado	
Incapacitado/a o invalidez permanente	
Jubilado/a	
Ama de casa	
Estudiante	
Otras posibilidades: ✍	
NS/NC	

P95. En su trabajo o en su último trabajo, ¿qué trabajo concreto hace (o hacía)? (Recoger el máximo de información)

____

P96. ¿Cuál es la actividad principal de la empresa donde trabaja o trabajaba? (Recoger el máximo de información)

____

P97. ¿Cuál es o cuál era su situación laboral en esa ocupación?

Asalariado	
Trabajador por cuenta propia, sin asalariados	
Trabajador por cuenta propia, con 10 o más asalariados	
Trabajador por cuenta propia, con menos de 10 asalariados	
Gerente de una empresa con 10 o más asalariados	
Gerente de una empresa con menos de 10 asalariados	
Capataz, Supervisor o encargado	
Otros (Becarios,…)	
NS/NC	

P98. ¿Cuál es el máximo nivel de estudios que ha alcanzado la persona principal? (No leer las alternativas de respuesta)

No sabe leer ni escribir	
No ha cursado estudios, pero sabe leer y escribir	
Estudios primarios incompletos (6o de EGB, ingreso o similar)	
Estudios primarios completos (EGB, grad. escolar, bachillerato elem. plan de antes BUP, FP1, “las 4 reglas”)	
Educación secundaria obligatoria (E.S.O)	
Estudios secundarios: BUP, COU, PREU, bachillerato plan nuevo	
FPII y ciclos formativos/ Fondos de garantía social	
Estudios universitarios de grado medio (diplomados)	
Estudios universitarios de grado superior (licenciados)	
Educación especial	
Otra posibilidad. Especificar: ✍	
NS/NC	

TODOS LOS SUJETOS

P99. En total, ¿cuántos metros cuadrados tiene su vivienda?

Metros	
NS/NC	

P100. ¿Cuántas habitaciones tiene su casa sin contar la cocina, el baño y el recibidor?

	Número de habitaciones
Con ventanas a la calle	
Con ventanas a patios interiores	
Sin ventanas	
Total	
NS/NC	

P101. Le queremos pedir si tendría algún inconveniente que de aquí a unos años nos volvamos a poner en contacto con usted en relación a temas de salud con tal de poder continuar con este estudio.

Sí, me parece bien que vuelvan a ponerse en contacto conmigo por este tema	
No, preferiría que no se vuelvan a poner en contacto conmigo por este tema	
NS/NC	

MUESTRA Y MEDIDAS

Accede a facilitarnos una muestra de saliva

Sí		
No		Motivo

	Sí		No
**Accede a que le tallemos**		Especificar en cm:	
**Talla autodeclarada**		Especificar en cm:	

	Sí		No
**Accede a que lo pesemos**		Especificar en kg:	
**Peso autodeclarado**		Especificar en kg:	
